# Anti-Metastatic and Anti-Inflammatory Effects of Matrix Metalloproteinase Inhibition by Ginsenosides

**DOI:** 10.3390/biomedicines9020198

**Published:** 2021-02-17

**Authors:** Sang Yeol Lee

**Affiliations:** Department of Life Science, Gachon University, San 65, Bokjeong-dong, Sujeong-gu, Seongnam-si 461-701, Gyeonggi-do, Korea; leesaye@gachon.ac.kr; Tel.: +82-31-750-8732

**Keywords:** matrix metalloproteinase, MAPK, ginsenosides, metastasis, inflammation

## Abstract

Matrix metalloproteinases (MMPs) are proteolytic enzymes which cleave extracellular matrix (ECM) and other substrates. They are deeply involved in both cancer metastasis and human chronic inflammatory diseases such as osteoarthritis and Crohn’s disease. Regulation of MMPs is closely associated with signaling molecules, especially mitogen-activated protein kinases (MAPKs), including three representative kinases, extracellular signal regulated kinases (ERK), p38 and c-Jun N-terminal kinases (JNK). Ginseng (*Panax* sp.) is a plant which has been traditionally used for medicinal applications. Ginsenosides are major metabolites which have potentials to treat various human diseases. In this review, the pharmacological effects of ginsenosides have been rigorously investigated; these include anti-metastatic and anti-inflammatory activities of ginsenosides associated with suppression of MMPs via regulation of various signaling pathways. This will highlight the importance of MMPs as therapeutic targets for anti-metastatic and anti-inflammatory drug development based on ginsenosides.

## 1. Introduction

Cancer metastasis is the primary cause of death of cancer patients, as it occupies about 67% of cancer deaths [1]. Cancer metastasis involves multiple cellular events: invasion into adjoining cells, migration and moving out to distant sites from the origin of tumorigenesis through the artery and lymphatic systems, and angiogenesis [2,3]. To promote metastasis, cancer cells have to break out and remodel the extracellular matrix (ECM) which is composed of collagen, laminins, fibronectin, elastin and multiple types of polysaccharide, because the ECM acts as a chemical and physical barrier anchoring cancer cells to the origin of tumorigenesis [4]. Therefore, ECM degradation has grabbed cancer researchers’ attention as one of the most critical events of metastasis. Matrix metalloproteinases (MMPs) enzymatically remove components of the ECM and allow cancer cells to migrate, invade and spread to tissues from remote organs [5]. It is previously reported that ECM alteration by MMPs can stimulate inflammation [6]. Furthermore, MMPs can process non-matrix proteins including inflammatory mediators, which indicates that they can also exacerbate inflammation and related human diseases [7]. Therefore, it is implicated that modulation of MMPs may contribute to improvement of cancer metastasis and inflammatory diseases.

**Figure 1:**
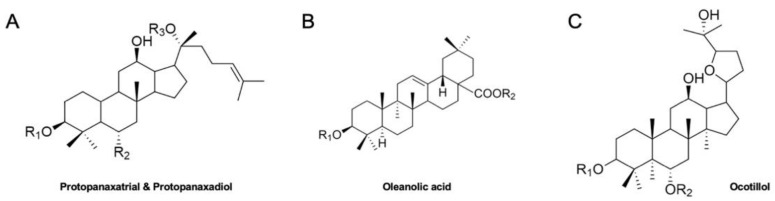
**Backbone structures of ginsenosides.** (**A**). Chemical structures of protopanaxatrial and protopanaxadiol, (**B**). Chemical structure of oleanolic acid, (**C**). Chemical structure of ocotillol.

Ginseng (*Panax* sp.) has been prevalently utilized for effective herbal medicine, dietary supplements and food products in Asian countries [8]. A broad range of clinical investigations have elucidated ginseng’s pharmacological effects [9,10]. Those effects include treatments of cancers, hypertension and diabetes, alleviation of stress, regulations of metabolism and cholesterol, and stimulation of physical performance [11]. Among its various constituents, ginsenosides are the main phytochemicals which play critical roles in the therapeutic effects of ginseng [12]. Ginsenosides have 4 major classes; protopanaxatrial and protopanaxadiol types which have dammarane backbone, oleanolic acid type with a pentacyclic triterpenoid, and ocotillol type which has a dammarane backbone and an epoxy ring at C20 position (Figure 1) [13]. The clinical potential of various ginsenosides has been reported for the treatment of chronic illnesses [14].

This review summarizes recent investigations into the suppressive effects of ginsenosides on cancer metastasis and inflammation, especially through the regulation of MMPs via modulation of various cellular signaling pathways. This will not only provide the mechanical basis of MMP inhibition on cancer metastasis and inflammation by ginsenosides but also raise the idea of developing new anti-metastatic and anti-inflammatory agents targeting MMPs based on the pharmacological effects of ginsenosides.

## 2. MMPs and Cancer Metastasis

During metastasis, cancer cells often undergo the epithelial into mesenchymal transition (EMT) which involves round-shaped epithelial cells changing into polarized mesenchymal cells to acquire mobility [15]. In addition, ECM degradation must be preceded to allow cancer cells “freedom” to move out. Once cancer cells are able to pass through the ECM, they migrate and invade into and out of blood/lymphatic vessels and reach out to tissues and organ systems in distance. In addition, angiogenesis also needs to be accompanied to provide oxygen and nutrients to fast-growing cancer cells.

**Figure 2:**
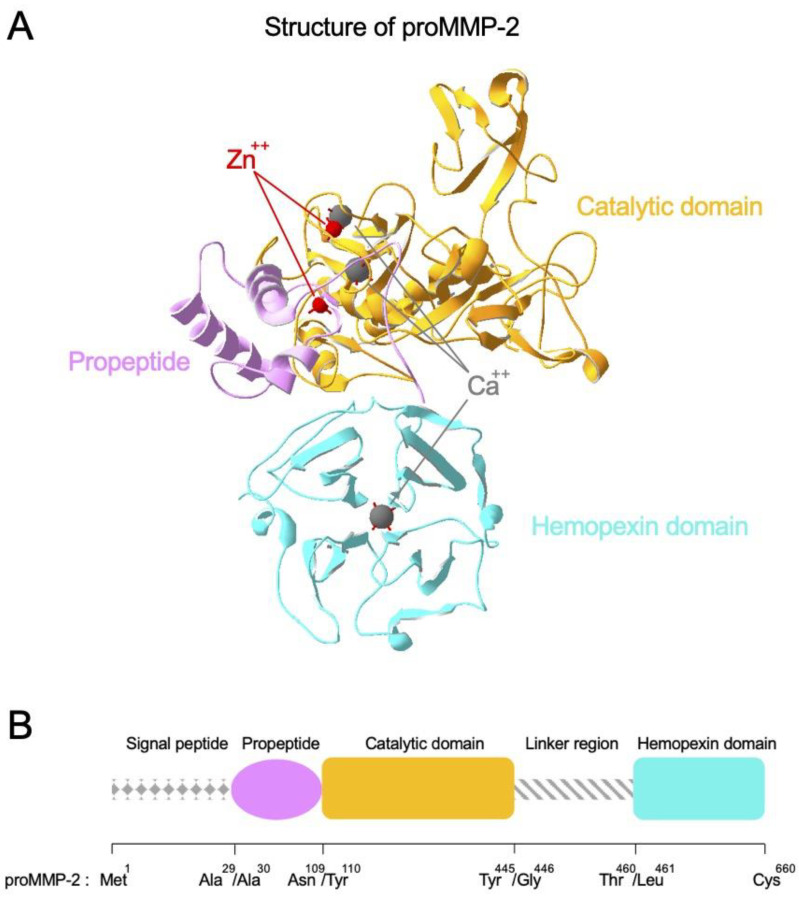
Structure and domain organization of proMMP-2. (**A**) Structure of proMMP-2 is presented as an example of MMP structure. proMMP-2 is composed of functionally distinct domains. The catalytic domain, propeptide and hemopexin domain are described in orange, pink and cyan color, respectively. Zn^++^ and Ca^++^ ions are indicated by red and gray color, respectively. Illustration is based on the crystal structure (PDB accession number: 1CK7). (**B**) Domain organization of proMMP-2 is presented as a representative example of MMP domain organization.

MMPs are a family of zinc-dependent multi-domain enzymes that play major roles in the degradation of ECM assembly (Figure 2A). Almost all MMPs show high homology in the structural organization as represented by proMMP-2 (Figure 2B). At amino-terminus, they have signal peptide that allocates the fate of MMPs whether they would be secreted or membrane-bound. Then, they have a propeptide region, a catalytic domain that possesses a catalytic zinc ion highly coordinated by calcium ions, and a long linker region. At carboxy-terminus, there is a hemopexin domain that forms homodimer for the full migratory ability in proMMP-9. Activation of proMMPs is achieved by proteolytic removal of the propeptide which physically blocks the zinc-containing active-site pocket. MMPs are categorized into several types based on their molecular structures and substrate preference (Table 1) [16,17,18,19]. For example, collagenases including MMP-1 and MMP-8 specialize in breakdown of interstitial collagens I, II and III, while gelatinases MMP-2 and MMP-9 mainly favor digestion of collagen type IV and gelatins. Since different types of MMPs can react with multiple constituents of the ECM, well-controlled expression and activity of MMPs is crucial for metabolic events of cells such as remodeling of tissues and cell development [20]. However, dysregulation of MMPs can result in severe human diseases such as inflammation and tumorigenesis. Considering that one of major roles of ECM assembly is acting as a physical barrier to protect cells from adverse effects, MMPs can promote aging of skin, arthritis and metastasis of cancer cells [21].

Functional roles of MMPs in cancer metastasis are not limited to the physical release from ECM structure. MMPs also have stimulatory or inhibitory effects on angiogenesis. Of many kinds of MMPs, MMP-2 and MMP-9 play the most important roles in angiogenesis process [22]. MMP-9 proteolytically digests ECM and releases vascular endothelial growth factor (VEGF), an ECM-bound factor that is important in the stimulation of angiogenesis. MMP-2 and MMP-9 also contribute to the angiogenesis via endostatin generation and angiostatin release by proteolytic cleavage of collagen XVIII and digestion of plasminogen, respectively [17]. MMP-9 also affects regulation of blood vessel development via degradation of type IV collagen of ECM and subsequent exposure of HUIV26 epitope. On the other hand, in the process of EMT, epithelial cells lose their integrity and undergo a phenotypic change into mesenchymal cells. By this process, cancer cell migration and invasion are stimulated. Elevated metastatic ability of mesenchymal cells would be associated with high expression level of MMPs in mesenchymal cells [23]. Moreover, previous report also indicated that MMPs promote cell migration non-enzymatically via hemopexin domain [24]. They demonstrated that the hemopexin domain of proMMP-9 contribute to the cell migration through the protein domain swapping approach. As mentioned, MMPs are associated with various steps of cancer metastasis and, thus, would be the prominent target for anti-cancer agent development.

## 3. MMPs and Inflammation

Inflammation is a crucial event to protect homeostasis against several stimuli including stress, injury and pathogens. However, aberrant inflammation results in severe human diseases such as diabetes, arthritis, cardiovascular diseases, neurodegenerative diseases, progression of cancers and fibrosis [13,25]. Inflammation frequently induces overexpression of MMPs which can aggravate cellular damage at inflammatory sites [7]. MMPs can regulate inflammation through cleaving non-matrix substrates including inflammatory cytokines and chemokines, which can be processed by MMPs to become active forms [7]. Association of MMPs with inflammation is reported by previous studies. For example, MMP-13 can promote inflammatory bowel disease in mice through cleavage of pro- tumor necrosis factor (TNF)-α to generate its mature form [26]. In addition, MMP-2, MMP-3, and MMP-9 can activate interleukin (IL)-1β via processing of its pro-forms [27]. Correlation of MMPs with inflammation was also found in clinical studies. For instance, elevated levels of MMP-2 and MMP-9 were observed in patients with Crohn’s disease and ulcerative colitis [28]. Clinical data of bronchiectasis patients also show that increase of MMPs are highly linked with inflammatory mediators [29]. Those studies suggest that appropriate regulation of MMPs may lead to alleviation and/or prevention of inflammation and related diseases.

## 4. Signaling Pathways Related with MMP Regulation

Expressions and activities of MMPs are regulated by various signaling pathways. Mitogen activated protein kinases (MAPKs), which includes three major classes Extracellular signal-regulated kinases (ERKs), p38 and c-Jun N-terminal kinases (JNKs), are a major family of enzymes highly associated with MMPs. Different stimuli, including growth factors and cytokines, can induce MAPK signaling pathways, and those signaling pathways commonly involve cascades consisting of more than three kinases acting sequentially to regulate target proteins via adding phosphate groups to serine and/or threonine residues [30]. Continuous phosphorylation leads to regulation of transcription factors which are often related with a wide range of cellular events. For instance, it is stated that suppression of the ERK and CREB pathway down-regulates transcriptional expressions of gelatinases (MMP-2 and MMP-9) in ovarian cancer cells [31]. Other reports highlight that external stimuli including ultraviolet (UV) and reactive oxygen species (ROS) can increase expression of MMP-1 via activation of MAPK signaling pathways [22,32]. In rat chondrocytes, IL-1β-induced collagen II degradation of MMP-1, MMP-3 and MMP-13 was regulated by ERK and p38 [33]. It is also documented that expression of various MMPs are regulated by p38 in bladder cancer (HTB5 and HTB9), breast cancer (MDA-MB231), hepatocellular carcinoma (SK-Hep1 and SNU-387), squamous cell carcinoma (UT-SCC7) and prostate cancer (PC3 and PC3-M) cell lines from patients with cancer [34,35,36,37,38]. JNK, another type of MAPK is closely linked with metastatic abilities in cancer cells from different organs including oral cancer and prostate cancer [39,40]. Strengthened cancer metastasis by JNK can accompany activation of MMPs. Modulation of JNK can down-regulate invasion of oral cancer cells via inhibited expression of MMP-2 and MMP-9 [39]. In prostate cancer, JNK activated by androgen receptor (AR) resulted in elevated MMP-9 levels and cell invasion [41].

Association of Phosphoinositide 3-kinase (PI3K)/ protein kinase B (Akt) signaling pathways with MMPs are also well known by several reports. In a previous paper, it is stated that lipopolysaccharide (LPS)-induced overexpression of MMP-9 in RBA-1 rat brain astrocytes was resulted from PI3K/Akt pathway [42]. Invasive abilities of PTK7-mediated TE-10 esophageal cancer cells could be enhanced by activation of PI3K/Akt/IKK/ nuclear factor κB (NF-κB) signaling axis along with ERK signaling pathways [43]. Akt activation is also needed for migration and invasion of SKOV-3 ovarian cancer cells, as the selective inhibition of the PI3K/Akt by LY294002 significantly suppressed expression of MMP-2 [44]. Other signaling proteins such as Smad, Notch and myeloid differentiation primary response 88 (MyD88) are also known to regulate expression of MMPs [45,46,47].

## 5. Ginseng and Human Chronic Diseases

It is reported that ginseng has been used for medication of human diseases about 5000 years ago in China [48]. There are 13 ginseng spices (*Panax ginseng* C. A. Meyer, *Panax japonicus* C. A. Meyer, *Panax pseudoginseng* Wallich, *Panax quinquefolius*, *Panax vietnamensis* Ha et Grushv., etc), which are cultivated in different countries and contain different constituents of metabolites (Table 2) [49]. Recent studies have investigated the potential of ginseng to treat a wide range of human diseases such as wrinkle formation, cold symptom complex, tumor progression, cancer-related fatigue, cardiovascular diseases, Alzheimer’s disease and inflammation, while improving glucose metabolism and cognitive skills [9,13,50,51,52,53,54]. Ginsenosides, major secondary metabolites purified from ginseng, possess therapeutic potential as well. For example, ginsenoside Rg3 has also shown to enhance survival of patients with lung, gastric and esophageal cancers in clinical studies [9]. Various in vitro and in vivo experiments demonstrate that other ginsenosides also exhibit anti-cancer and anti-inflammatory effects, although more clinical trials are needed to confirm those positive outcomes [9,13].

## 6. Anti-Metastatic and Anti-Inflammatory Effects of Ginsenosides via Regulation of MMPs

As mentioned above, ginsenosides have been reported to have suppressive effects on varied human diseases. Of note, anti-metastatic and anti-inflammatory effects of ginsenosides are also well described. Furthermore, those effects are intimately linked with the inhibition of expression and/or enzymatic activities of MMPs via modulation of signaling pathways. Various in vitro and in vivo assays highlight that ginsenosides including compound K (CK), Rg1, Rg3, Rh1, Rh2 and Rd reduced metastatic abilities of various tissue-specific cancer cells by down-regulating the transcriptional expressions of several MMPs (MMP-1, MMP-2, MMP-3, MMP-7, MMP-9 and MMP-13). In addition, ginsenosides exerted anti-inflammatory activities in cells or animals with a model of inflammatory diseases. Synergistic MMP-inhibiting effects of some ginsenosides combined with other reagents are also documented. This suggests the possibility of ginsenosides being developed to avoid multidrug resistance, one of the difficulties in developing a new therapeutic agent [63]. In this part of the review, I will summarize the effects of representative ginsenosides on MMPs.

### 6.1. Rg1

**Figure 3:**
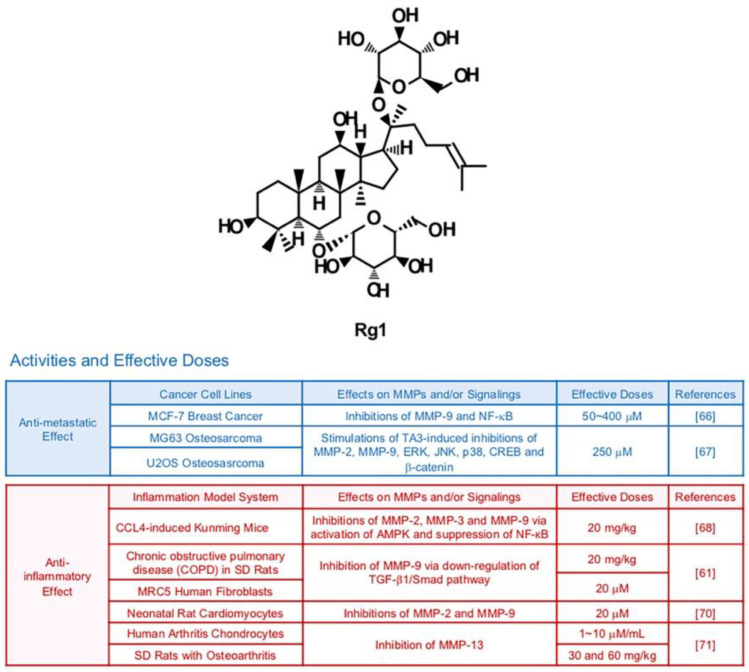
Summary of activities of ginsenoside Rg1. Structure of Rg1 and its effects on MMPs and/or signalings [61,66,67,68,70,71].

Rg1 can be isolated from the root or stem of Panax ginseng (Figure 3). Several studies demonstrate the pharmacological effects of Rg1 in cells from different organ systems including the nervous and immune systems [64,65]. Rg1 (50, 100 and 200 μM) repressed phorbol myristate acetate (PMA)-induced metastatic abilities of breast cancer cells via down-regulated DNA binding activity of NF-κB and reduced expression of MMP-9 [66]. Furthermore, Rg1 can exert synergistic MMP-inhibiting effects with other drugs. A recent study showed that Rg1 could intensify anti-metastatic effects of Timosaponin AIII, an anti-cancer steroid saponin, in MG63 and U2OS osteosarcoma cells [67]. In particular, gelatin cleavage by MMP-2 and MMP-9 were remarkably reduced via transcriptional regulation when cells were exposed to both Timosaponin AIII and Rg1. Repressive effects of Timosaponin AIII (6 μM) on JNK, p38 and ERK were significantly strengthened by the combination with Rg1 (250 μM) [67].

MMP-inhibiting and anti-inflammatory effects of Rg1 from several tissues are also demonstrated. Yao et al. reported that Rg1 dissipated the elevated expression of MMP-2, MMP-3 and MMP-9 in CCl_4_-exposed inflammatory liver from Kunming mice (20 mg/kg) via activation of AMPK and suppression of NF-кB [68]. Rg1 (20 mg/kg) relieved ECM degradation by MMP-9 which contributes to cigarette smoke-induced pulmonary fibrosis in Sprague-Dawley (SD) rats (20 mg/kg) and MRC5 human fibroblasts (40 μM) through down-regulation of TGF-β1/Smad pathway [69]. Rg1 could diminish inflammation of rat cardiomyocytes (20 μM) by decreased gelatinases (MMP-2 and MMP-9) [70]. MMP-13 was down-regulated by Rg1 in human arthritis chondrocytes (10 μg/mL) and SD rats with osteoarthritis (OA) (30 mg/kg) as well [71].

### 6.2. Rg3

**Figure 4:**
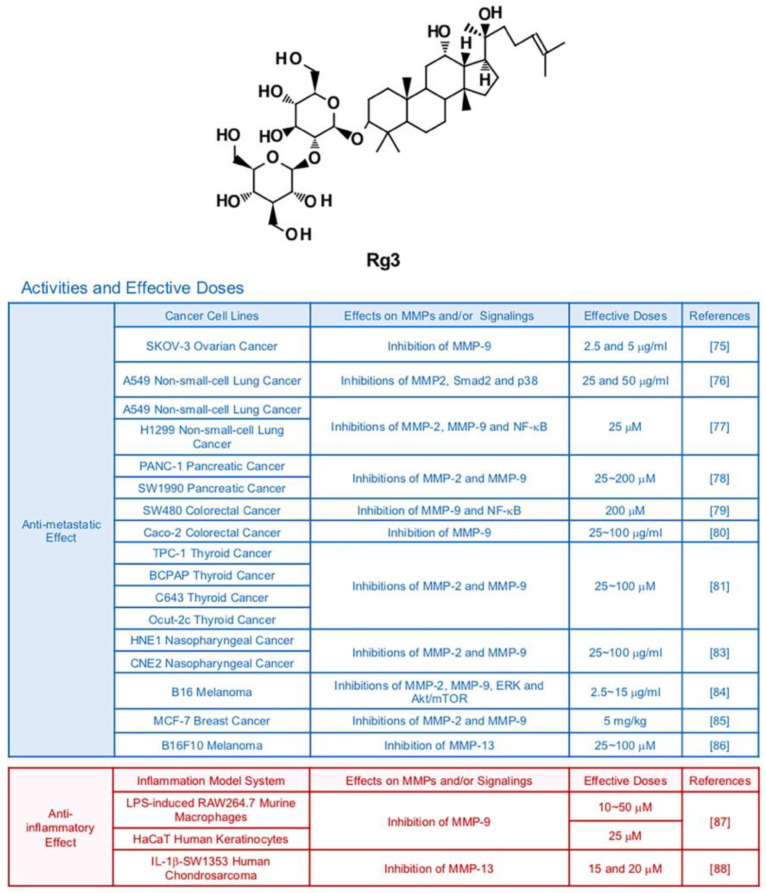
Summary of activities of ginsenoside Rg3. Structure of Rg3 and its effects on MMPs and/or signalings [75,76,77,78,79,80,81,83,84,85,86,87,88].

Ginsenoside Rg3 is one of major compounds found in red ginseng which is a root of steamed *Panax ginseng* C. A. Meyer [72] (Figure 4). While Rg3 has a potential to display various therapeutic effects, it especially strengthened anti-cancer efficacy compared with other ginsenosides with its strong pro-apoptotic activities [73]. As mentioned earlier, clinical trials indicate that Rg3 can be developed as an effective anti-cancer drug [9]. The major anti-tumor effects of Rg3 were suppression of proliferation, metastasis, angiogenesis, and promotion of apoptosis [74]. Various studies revealed that Rg3 diminished the metastatic abilities of cancers via inhibition of MMPs. Several in vitro and in vivo studies reported that Rg3 impeded migration and invasion of ovarian (SKOV-3), lung (A549, H1299 and LLC1), pancreatic (tissues from cancer patients), colorectal (SW480 and Caco-2), thyroid (TPC-1, BCPAP, C643, and Ocut-2c), nasopharyngeal (HNE1 and CNE2), skin (B16), and breast (MCF-7) cancers via down-regulation of MMP-2 and/or MMP-9 [75,76,77,78,79,80,81,82,83,84,85]. It is notable that 20(R)-Rg3, not 20(S)-Rg3 showed inhibitory effects in A549 lung cancer cells, indicating that stereospecificity should be considered in some contexts [76]. also hampered metastatic activities of B16F10 melanoma cells through reducing expression of MMP-13 [86]. Of note, MMP inhibition by Rg3 in melanoma and lung cancer cells is highly linked with inactivation of MAPKs and/or Akt signaling pathways [76,84]. Synergistic effects of Rg3 with other compound on MMP expression were also reported. Combined treatment with Rg3 (5 mg/kg) and the anti-angiogenic drug Endostar (5 mg/kg) inhibited transcription of MMP-2 and MMP-9 in mice bearing MCF-7 breast tumors, notably better than treatment with Endostar alone [85].

In regard to inflammation, Rg3 attenuated protein expression of MMP-9 in LPS-induced RAW264.7 murine macrophages and non-stimulated HaCaT human keratinocytes (50 μM and 25 μM, respectively) while not affecting cell viability [87]. Moreover, Rg3 (10, 15 and 20 μM) inhibited MMP-13 expression in IL-1β-SW1353 human chondrosarcoma cells [88].

### 6.3. Rh1

**Figure 5:**
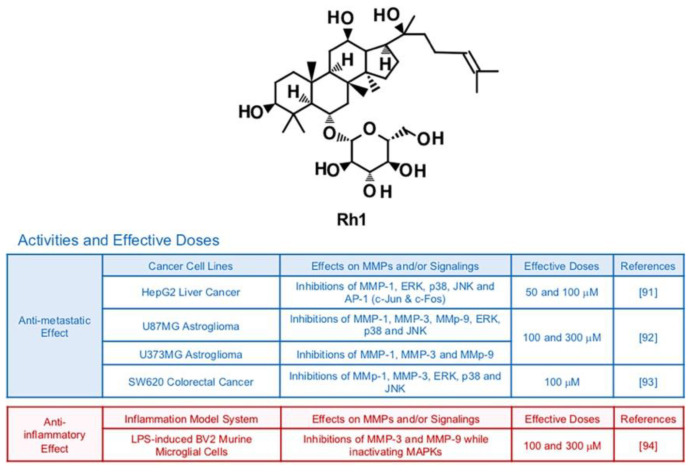
Summary of activities of ginsenoside Rh1. Structure of Rh1 and its effects on MMPs and/or signalings [91,92,93,94].

Ginsenoside Rh1, a metabolite from Rg1, can be isolated from red ginseng which is a root of steamed *Panax ginseng* C. A. Meyer [89] (Figure 5). Previous investigations reported Rh1′s potential to protect neuronal cells, inhibit neoplasm and improve chronic inflammatory diseases [90]. Phosphorylation of MAPKs including ERK, JNK and p38 was notably down-regulated by treatment with Rh1 (50 μM and 100 μM) in HepG2 liver cancer cells [91]. This modulation is highly correlated with suppressed activity of AP-1 transcription factors and reduced collagenase activity of MMP-1 [91]. In vitro experiments showed that Rh1 (100 μM and 300 μM) effectively inactivating MAPKs, AP-1 and NF-κB of U87MG astroglioma cells, resulting in impeded cell migration and invasion [92]. Metastatic abilities of SW620 colorectal cancer cells were also suppressed by Rh1 (100 μM) via inhibition of MAPK signaling transduction pathways and transcriptions of MMP-1 and MMP-3 while increasing expression of tissue inhibitor of metalloproteinases 3 (TIMP3), a negative regulator of MMPs [93]. Rh1 could effectively protect cell death and inhibit transcriptional expression of MMP-3 and MMP-9 with other pro-inflammatory cytokines in LPS-induced BV2 murine microglial cells (100 and 300 μM) while inactivating MAPKs [94].

### 6.4. Rh2

**Figure 6:**
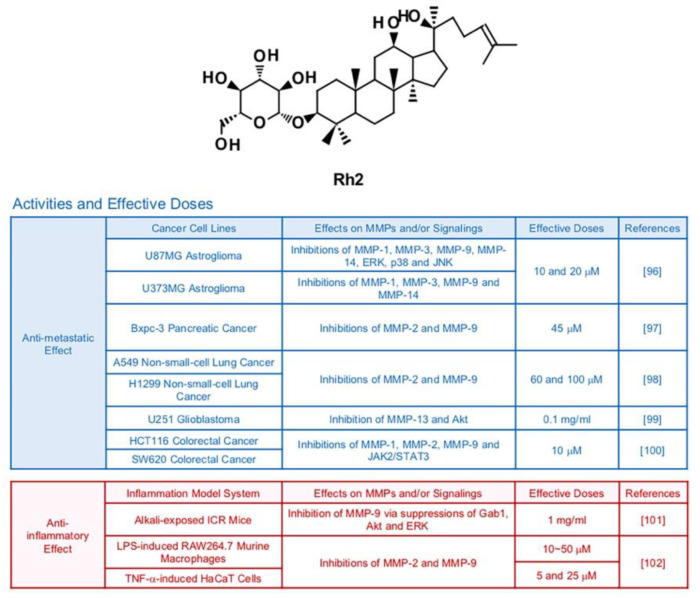
Summary of activities of ginsenoside Rh2. Structure of Rh2 and its effects on MMPs and/or signalings [96,97,98,99,100,101,102].

Earlier studies demonstrated that the ginsenoside Rh2 prompted cancer apoptosis through activation of cell death signaling pathways that start from stimulation of death receptors in mitochondria or cell membranes [95] (Figure 6). Rh2 has potential to effectively suppress growth of metastasis of cancer cells as well. In one research, Rh2 (10 and 20 μM) repressed the transcriptions of MMP1, MMP-3, MMP-9 and MMP-14 in U87MG and U373MG human astroglioma cells [96]. In that study, inhibition of MAPKs, AP-1 and NF-κB were observed in U87MG human astroglioma cells after treatment with Rh2 [96]. Rh2 also diminished metastatic activities of pancreatic (Bxpc-3 at a dose of 45 μM) and lung (A549 at a dose of 60 and 100 μM) cancer cells via inhibition of MMP-2 and MMP-9 [97,98]. In U251 glioma cancer cells, Rh2 (0.1mg/mL) displayed anti-metastatic effects mediated by regulation of Akt signaling pathways and reduced MMP-13 expression [99]. Treatment with Rh2 (10 μM) hindered expression of three types of MMPs, MMP-1, MMP-2 and MMP-9 in HCT116 and SW620 human colorectal cancer cells via regulation of JAK2 and STAT3 pathways [100].

A previous in vivo study stated that treatment with Rh2 (1 mg/mL, 5 times a day) suppressed Grb-2–associated binder 1 (Gab1), Akt and ERK, which resulted in lowered expression of MMP-9 and corneal neovascularization in alkali-exposed ICR mice [101]. 20(R)-Rh2, which is a minor stereoisomer of Rh2, exerted anti-inflammatory and MMP-inhibiting effects in LPS-induced RAW 264.7 cells and TNF-α-induced HaCaT cells [102].

### 6.5. Rb1

**Figure 7:**
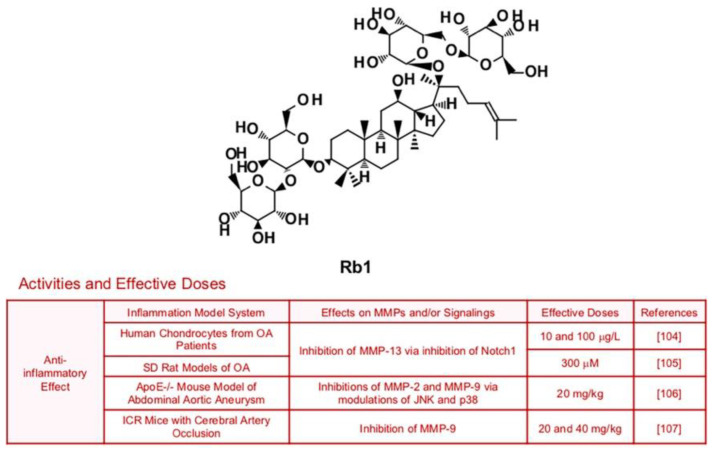
Summary of activities of ginsenoside Rb1. Structure of Rb1 and its effects on MMPs and/or signalings [104,105,106,107].

Ginsenoside Rb1, which has multiple biological activities such anti-oxidant and anti-inflammatory effects, could suppress expression of collagenase MMP-13 in SW1353 human chondrosarcoma cells and SD rats with anterior cruciate ligament transection (80 µM), via inhibition of Notch molecular signaling pathways [103] (Figure 7). Since this study used chondrosarcoma cells, it is expected that Rb1 may also repress cancer metastasis. Rb1 also improved inflammation and MMP-13 expression in human chondrocytes from OA patients and SD rat models of OA [104,105]. Rb1′s anti-inflammatory potential is not limited to OA, as Rb1 ameliorated vascular disease and brain damage associated with inflammation. Administration of Rb1 (20 mg/kg) successfully reduced expression of MMP-2 and MMP-9 via modulation of JNK and p38 pathways, which led to ECM destruction and vascular remodeling in ApoE^−/−^ mouse model of Abdominal aortic aneurysm (AAA) [106]. Furthermore, treatment with Rb1 (20 and 40 mg/kg) prevented blood-brain barrier through inhibition of pro-inflammatory mediators including MMP-9 [107].

### 6.6. Compound K (CK)

**Figure 8:**
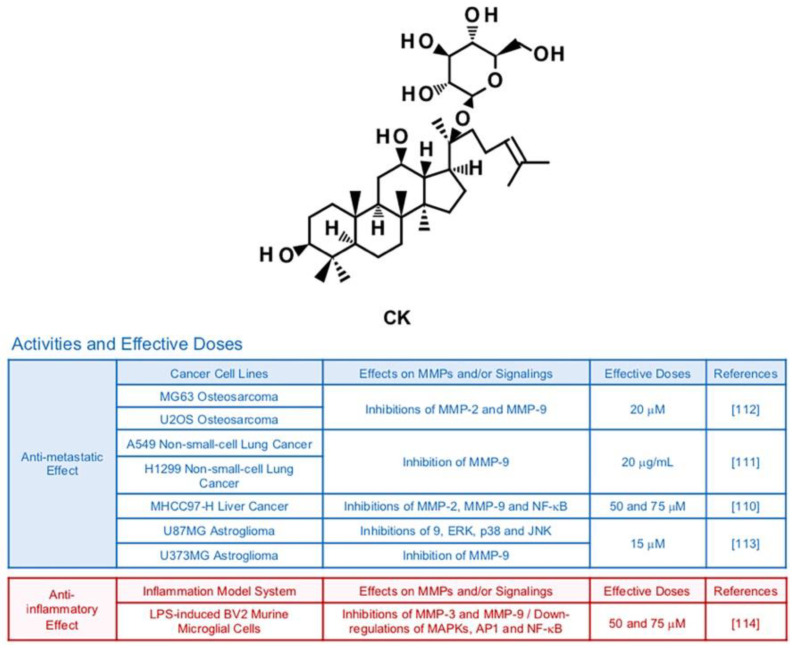
Summary of activities of ginsenoside CK. Structure of CK and its effects on MMPs and/or signalings [110,111,112,113,114].

Compound K (CK) is a ginsenoside originated from Rb1, a main compound of *Panax ginseng* C. A. Meyer [108] (Figure 8). There have been in vitro and in vivo studies emphasizing the therapeutic potency of CK in improvement of allergies, diabetes, inflammation, skin aging and hepatocellular damage [109]. MMP-inhibiting effects of CK are also reported by several publications. In MHCC97-H hepatocellular carcinoma cells, CK treatment (50 and 75 μM) inhibited expression of MMP-2 and MMP-9 via reduced activity of NF-κB [110]. CK mixed with miscells (20 μg/mL) successfully lowered protein levels of MMP-9 in A549 lung cancer cells, resulting in declined migration and invasion in mice bearing A549 cells [111]. Invasive and migratory abilities of MG63 and U2OS osteosarcoma cells decreased due to weakened PI3K, Akt, mTOR and p70S6K1 signaling pathways and lessened expression of gelatinases by CK (20 µM) [112]. Furthermore, CK (15 µM) effectively restrained expression of MMP-9 in U87MG astroglioma cells via suppression of ERK, JNK, and p38 MAPKs [113]. CK attenuated expression levels of MMP-3 and MMP-9 in LPS-induced BV2 murine microglial cells (50 and 75 μM), while down-regulating MAPKs, AP-1 and NF-κB [114].

### 6.7. Rd

**Figure 9:**
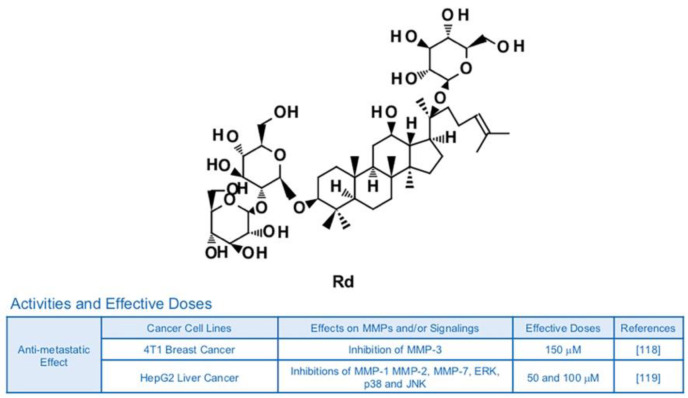
Summary of activities of ginsenoside Rd. Structure of Rd and its effects on MMPs and/or signalings [118,119].

The ginsenoside Rd is known to possess neuroprotective potential, as it can inhibit activity of NF-κB and expression of TNF-α in LPS-induced N9 microglial cells [115] (Figure 9). Rd also showed anti-inflammatory effects in rats with ischemia via reduced protein levels of inducible nitric oxide synthase (iNOS) and cyclooxygenase (COX)-2 [116]. Additionally, Rd attenuated the generation of nitric oxide, production of prostaglandin E2 (PGE2) and the DNA binding ability of NF-κB, leading to a decrease in inflammatory responses of RAW264.7 murine macrophage cells [117]. Although there is no publication focusing on the relationship with Rd, inflammation and MMPs yet, inhibitory effects of Rd on anti-metastatic effects and MMPs are reported. Anti-metastatic effects of Rd (150 μM) on 4T1 breast cancer were achieved by down-regulation of MMP-3 via regulation of Smad2 [118]. In HepG2 human liver cancer cells, Rd (50 and 100 μM) reduced phosphorylation of MAPKs and DNA binding activity of AP-1 in addition to leading to dwindled expression of collagenase MMP-1, gelatinase MMP-2 and matrilysin MMP-7 [119].

### 6.8. Other Ginsenosides

**Figure 10:**
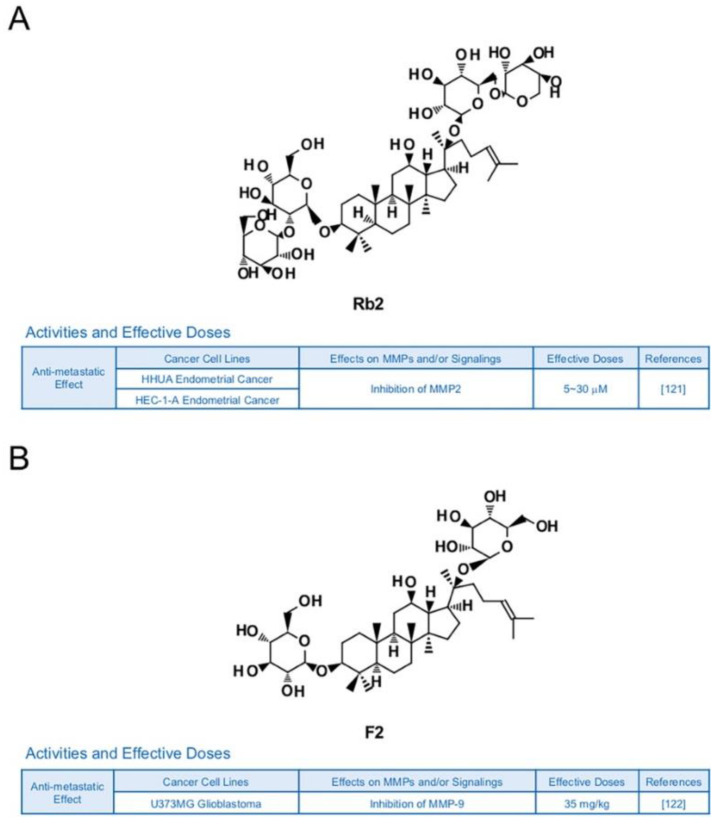

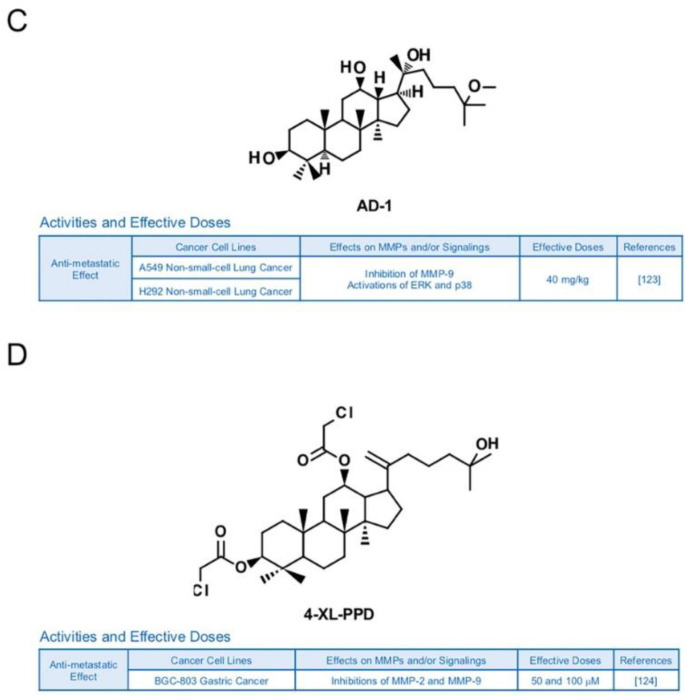
Summary of activities of ginsenosides Rb2, F2, AD-1 and 4-XL-PPD. Structures of Rb2 (**A**), F2 (**B**), AD-1(**C**) and 4-XL-PPD (**D**) and their effects on MMPs and/or signalings [121,122,123,124].

Other types of ginsenosides also have the potential to suppress MMPs. Ginsenoside Rb2 was reported to lower the accumulation of hepatic lipid in obese mice, blood glucose level in rats with diabetes and triacylglycerol in 3T3-L1 adipocytes [120]. In previous research, Rb2 inhibited expression of MMP-2 and invasive activities in HHUA and HEC-1-A endometrial cancer cells [121] (Figure 10A). Ginsenoside F2 (35 mg/kg), which can be derived from varied types of protopanaxadiol saponins, is known to induce apoptosis and repress invasive abilities of U373MG glioblastoma cells-implanted SD rats with reduced expression levels of MMP-9 [122] (Figure 10B). Novel ginsenoside derivatives AD-1 (40 mg/kg) and 4-XL-PPD (50 and 100 μM) are also reported to inhibit protein levels of MMP-9 and/or MMP-2 in athymic nude mice with A549 lung cancer cells and MGC-803 human gastric cancer cells, respectively [123,124] (Figure 10C,D).

**Figure 11:**
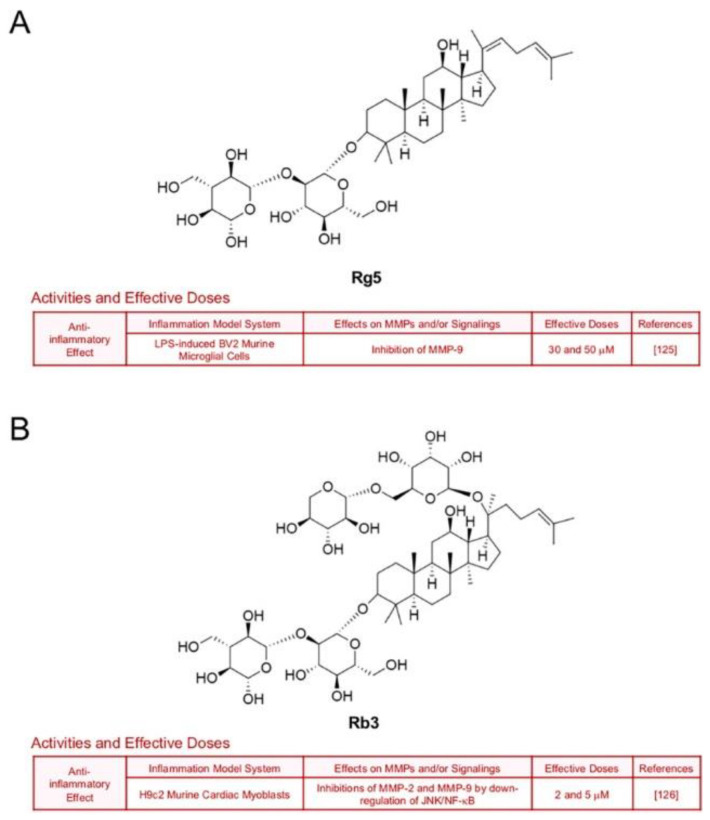

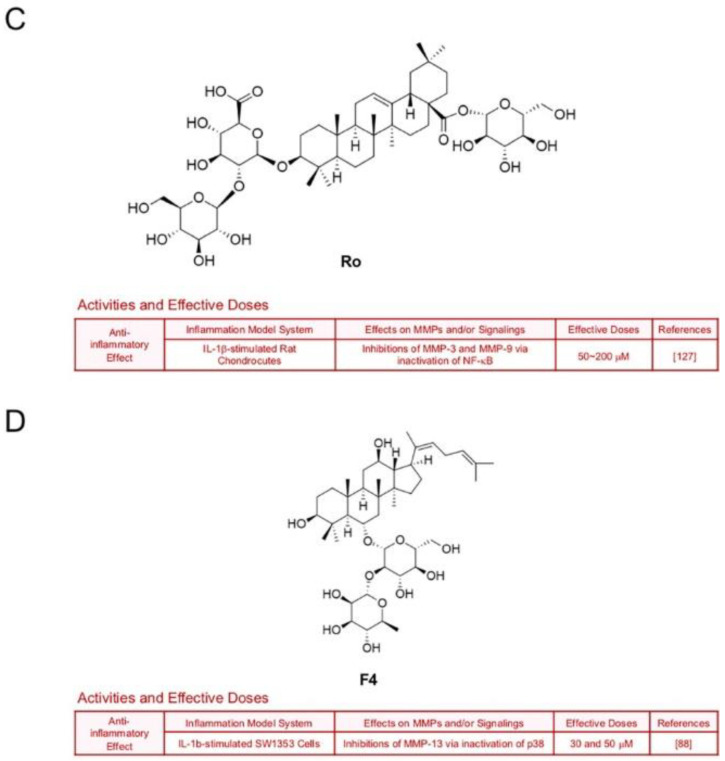
Summary of activities of ginsenosides Rg5, Rb3, Ro and F4. Structures of Rg5 (**A**), Rb3 (**B**), Ro (**C**) and F4 (**D**) and their effects on MMPs and/or signalings [88,125,126,127].

Treatment with ginsenoside Rg5, one of main components in steamed ginseng, decreased LPS-induced transcription of MMP-9 in BV2 microglial cells (30 and 50 μM), which implies a neuroprotective potential [125] (Figure 11A). Ginsenoside Rb3 also hindered expression of gelatinases by down-regulation of JNK/NF-кB signaling axis in H9c2 murine cardiac myoblasts which were exposed to oxygen and glucose deprivation [126] (Figure 11B). Ginsenoside Ro, an oleanolic acid type-ginsenoside, inhibited inflammation and protein levels of MMP-3 and MMP-9 through inactivation of NF-кB in IL-1β-stimulated rat chondrocytes [127] (Figure 11C). Additionally, ginsenoside F4 (30 and 50 μM) diminished MMP-13 expression in IL-1β-stimulated SW1353 cells through inactivation of p38 [88] (Figure 11D).

## 7. Conclusions and Perspectives

In conclusion, ginsenosides’ potential to exert anti-metastatic and anti-inflammatory effects has a high association with regulation of MMPs and related signaling pathways. Even though there are numerous molecules proposed to be targets for suppressing metastatic abilities of cancers and inflammatory diseases, MMPs are one of the most intriguing ones because both their ECM degradation and non-enzymatic modes of action considerably contribute to metastasis and immune responses. Since ginsenosides’ MMP suppression had synergy with a few known compounds, co-treatment with other chemicals including MMP inhibitors may also exhibit promising results in the future [67,85,108,128]. This escalates the value of ginsenosides as promising anti-cancer and anti-inflammatory agents to be further developed and modified. However, there is a limitation that ginsenosides do not have sufficient clinical studies to ensure their potential yet, as pointed out in a previous review [9]. One of the main reasons is their low solubility and bioavailability which are important for oral administration [129,130]. Although several positive outcomes were shown from in vivo studies, effective methods such as chemical modifications and delivery with micro/nano particles may needed to enhance the parameters of bioavailability (absorption, metabolic rate and efflux of ginsenosides) to obtain more consistent experimental data and increase the possibility of clinical applications [131,132]. Furthermore, it should be considered that ginsenosides are highly likely to affect not only MMPs, but also quite a few other proteins. It is implicated that use of ginsenosides for targeting MMPs may give rise to unexpected results by changes of other unknown factors. More thorough in vitro and in vivo assays are required to clarify the correlation between ginsenosides, MMPs and other related molecules for drug development.

## Figures and Tables

**Table 1:** Classification of major MMPs and their substrate specificity for ECM components.

Group	MMP	ECM Substrates
Collagenases	MMP-1	Collagens (III>I>II, VII, X), gelatin, entactin, laminin, aggrecan, perlecan
	MMP-8	Collagens (I>II>III, VII, X), gelatin, fibronectin, aggrecan
	MMP-13	Collagens (II>III>I, VII, X), gelatin, aggrecan, entactin
Gelatinases	MMP-2	Gelatin, collagen (IV-VI), fibronectin, elastin
	MMP-9	Gelatin, collagen (IV, V, VII, X, XIV, XVIII), fibrillin, elastin, osteonectin, fibronectin, elastin
Stromelysins	MMP-3	Gelatin, collagens (II, III, IV, V, IX, X, XI, XVIII), laminin, fibronectin, aggrecan, fibrin, elastin, perlecan
	MMP-10	Collagen (I, III-V), gelatin, elastin, aggrecan, proteoglycan
	MMP-11	Fibronectin, gelatin, laminin, aggrecan
Matrilysins	MMP-7	Collagen (IV–X, XVIII), gelatin, laminin, aggrecan, fibronectin, vitronectin, fibrin, entactin, vitronectin
	MMP-26	Gelatin, collagen type IV, fibronectin, fibrin
Metalloelastase	MMP-12	Elastin, collagen (I, IV, XVIII), gelatin, laminin, vitronectin, fibronectin, proteoglycan
Membrane-type MMPs	MMP-14	Gelatin, collagens (I, II, III), fibronectin, laminin, fibrin, perlecan
	MMP-15	Collagens (I, IV), perlecan, fibronectin, laminin, aggrecan, perlecan
	MMP-16	Fibrin, gelatin, type III collagen, fibronectin, vitronectin, laminin
	MMP-17	Gelatin, fibrin
	MMP-24	Gelatin, fibronectin, laminin, proteoglycans

**Table 2:** Ginseng species and their major ginsenosides.

Ginseng Species	Major Ginsenosides	References
*Panax ginseng* C. A. Meyer	Rb1, Rb2, Rb3, Rc, Rd, Re, Rg1, Rh1	[55,56,57]
*Panax japonicus* C. A. Meyer	Rb1, Rc, Re, Rg1, R1, R2	[58]
*Panax pseudoginseng* Wallich	Rg1, Re, Rb1, Rc, Rb2, Rd	[56]
*Panax quinquefolius*	Rb1, Re, Rd, Rg1, Rc, Rb2	[56,59]
*Panax vietnamensis* Ha et Grushv.	Rb1, Rc, Rb2, Rd	[56]
*Panax stipuleanatus* H. T. Tsai and K. M. Feng	Rb1, Rb3, Rc, Rd	[60]
*Panax trifolius* L.	Ro, Re, Rf, Rg2	[61]
*Panax zingiberensis* C. Y. Wu and K. M. Feng	Ro, Rg1, Rh1	[62]
*Panax wangianus* Sun	Unknown	
*Panax major* Ting	Unknown	
*Panax omeiensis* J. Wen	Unknown	
*Panax sinensis* J. Wen	Unknown	
